# Investigating the Impact of Institutions on Small Business Creation Among Saudi Entrepreneurs

**DOI:** 10.3389/fpsyg.2022.897787

**Published:** 2022-06-13

**Authors:** Ali Saleh Alshebami, Abdullah Hamoud Ali Seraj

**Affiliations:** ^1^Applied College, King Faisal University, Al Hofuf, Saudi Arabia; ^2^Department of Management, College of Business Administration, King Faisal University, Al Hofuf, Saudi Arabia

**Keywords:** Saudi Arabia, SMEs, entrepreneurship, institutional factors, entrepreneurs

## Abstract

Institutions significantly impact people’s attitudes and behaviors, both favorably and negatively. The purpose of this article is to examine the influence of several institutions on the intentions and decisions of Saudi entrepreneurs to start a business. Accordingly, the study on which this article is based used cross-sectional data of 3,376 respondents obtained from the Global Entrepreneurship Monitor (GEM) in 2016. The findings demonstrated that insufficient business legislations and policies have a detrimental impact on the ability to start small businesses. Furthermore, it was discovered that the more media attention is given to successful entrepreneurs, the greater the likelihood of small businesses being established. Surprisingly, the typically high regard for successful entrepreneurs had no positive impact on the establishment of small businesses in Saudi Arabia. Moreover, there was no negative impact of fear of failure on the likelihood of starting small firms. Finally, the influence of control variables, such as age and gender, was also varied. Because the study was limited to the context of Saudi Arabia, future research could focus on expanding the analysis to other Gulf countries and including more institutions.

## Introduction

Entrepreneurial activities have been viewed as an effective driver of economic development and expansion in a large body of literature in the field of entrepreneurship (Minniti, 2008; Ali and Hilman, 2020; Aloulou, 2021; Cai et al., 2021; Jiatong et al., 2021). This is due to the potential of entrepreneurial activities to facilitate firms’ growth and innovation and to increase people’s wealth (Chew et al., 2021). Entrepreneurship is a process that involves seizing an opportunity, exerting effort, taking risks and being competitive (Miller, 1983) to establish a new firm or improve an existing one. Entrepreneurship leads to the generation of new job possibilities and the reduction of poverty in the long run (Alshebami et al., 2020). Consequently, there has been an ongoing global and local effort to assist small and medium-sized enterprises (SMEs) and promote entrepreneurship because of their critical role in economic development (Gilmore et al., 2013).

Furthermore, despite the tremendous effort put into entrepreneurship, it has mostly focused on the impact of external criteria alone on the possibility of establishing a business (age, gender, income, etc.). Other subjective aspects, such as individual intentions and perspectives concerning the cultural, political, demographic and environmental contexts of entrepreneurship, should be investigated as well, as they have a role in entrepreneurial activities (AbuBakar et al., 2017). In short, institutional factors and their impact on entrepreneurship in particular, as well as economic growth in general, must be examined (Urbano et al., 2018).

Institutions are thought to be regulators of people’s conduct and attitudes in general. According to North (1991), institutions are of two types—namely formal and informal institutions. Formal institutions are defined as those formal rules, laws, constitutions and official regulations that individuals should follow. On the other hand, informal institutions are defined as informal constraints, such as taboo, customs and codes of conduct. These institutions have also been classified by Scott (1995) into three dimensions: regulative, normative and cultural-cognitive. The regulative dimension represents formal institutions, and the normative and cultural-cognitive dimensions represent informal institutions. Although these two classifications of institutions have different categorizations, they complement each other (Peng et al., 2009). Institutions, or so-called “social environment elements,” influence people’s decision about whether to establish a business and become entrepreneurs (Bruton et al., 2010)—not only that, they also influence the characteristics of the firms intended to be established (Fuentelsaz et al., 2015). Institutions are referred to as game rules because they arrange various forms such as economic, political and social ties, resulting in better networks (North, 1990). They also play a crucial role in discouraging or encouraging individuals to pursue opportunities in the market (Welter and Smallbone, 2008). Both formal and formal institutions exert different effects on individuals and their businesses. For example, in the extant literature, it has been found that formal institutions influence the business establishment (Urbano et al., 2018). On the other hand, informal institutions also influence entrepreneurial activities (Kreiser et al., 2010; Wennberg et al., 2013).

In this study, we considered the classification of Scott (1995) for defining institutions according to their regulative, normative and cultural-cognitive dimensions. We investigated how these institutions are linked to the nascent stage of entrepreneurial activities in the context of Saudi Arabia. This is because Saudi Arabia is an oil-rich country, and recently there has been a fluctuation in global oil prices, leading to a budget deficit in the country. Consequently, the government established plans and tactics to diversify its economy and income to meet continuous economic challenges. As a result, Saudi Vision 2030 was created to implement major economic reforms. The vision recognized the issues faced by the Saudi economy, especially the SME sector, and particularly the complexity and length of administrative procedures involved in starting a business. Thus, the effect of these institutional factors must be explored. Saudi Vision 2030 is intended to enhance the contribution of the SME sector to the national GDP from 20 to 35% by 2030 (Alamoudi and Bagaaifar, 2017; Khan and Alsharif, 2019) and to reduce the country’s unemployment rate from 11.6 to 7%. It also aims to support roughly 950,000 Saudi SMEs operating in the market since 2017. These SMEs are categorized as micro enterprises, employing about 1 million Saudis. Therefore, to encourage and help Saudi citizens to create small businesses and become entrepreneurs, the Saudi government has established incubators, initiatives and technical and financial organizations (Alshebami et al., 2020). These include, for example, Monshaat, the Human Resource Development Fund, Wadi Makkah, the Centennial Fund, Moden, King Abdul-Aziz City for Science and Technology, and others. However, and despite the support provided by the government, many challenges continue to be faced by the SME sector. For example, according to a study by Khan and Alsharif (2019), a significant barrier that individuals who want to start a business face are difficulties such as electricity tariffs, government regulations and licenses.

Furthermore, other literature concerning Saudi Arabia and its environmental factors reported different results. For example, the presence of official institutions may serve as a strong motivator for supporting women entrepreneurs, while the presence of lack of saving culture, may negatively impact business establishments since they are unable to save and subsequently reinvest (Alshebami and Seraj, 2021b). Additionally, a study by AbuBakar et al. (2017) also examined the effect of institutions on the propensity to start small enterprises in Saudi Arabia and reported a different effect on entrepreneurship. However, they employed GEM data from 2009, which is considered outdated, taking into account the various reforms conducted in the country since 2009. In another article, by Alshebami and Seraj (2021a), It was disclosed that having a connection with other entrepreneurs, recognizing opportunities in the market, and having experience in business will all support individuals to become entrepreneurs. Aljarodi (2020), on the other hand, reported that the more female entrepreneurs are encouraged to launch new businesses due to favorable policies, the more such businesses are actually established. Moreover, a study by Albihany (2019) examined the role of Wasta (an informal institution) on Saudi early-stage entrepreneurship and reported significant results. Aloulou (2021) also found a substantial correlation between characteristics of the institutional context, students’ perceived desire to launch a new business, and the feasibility of entrepreneurial behavior. Other research has focused on various facets of entrepreneurship. Therefore, the above review demonstrates that the literature on the impact of institutional determinants on small business creation in Saudi Arabia is still limited (Farid et al., 2011; Aloulou, 2020). Accordingly, this study explored several elements from the regulative, normative and cultural-cognitive dimensions and their impact on small venture creation in Saudi Arabia. The following questions were addressed:

1. Does the provision of suitable business regulations and policies, media attention, and the high status of entrepreneurs in Saudi Arabia assist in establishing small businesses?
2. Does the fear of failure deter people in Saudi Arabia from launching small businesses?

The remainder of this article is divided as follows. The next section reviews the relevant literature and formulates the hypotheses, after which, in the following section, the research approach is discussed. In the fourth section, the study’s results are reported, which is followed by a discussion of the results in the fifth section. Lastly, the final section covers the limitations of the research and provides recommendations for future research.

## Literature Review and Hypothesis Development

The research, based on institutional theory, divided institutions into three dimensions: regulative, normative and cultural-cognitive (Scott, 1995). The regulative dimension shows that individuals behave according to official laws and regulations, which are enacted by formal institutions. In the context of the present work, emphasis was placed on the provision of the necessary legislative support and policies to encourage the formation of new businesses. The normative dimension, on the other hand, includes what is desirable in a society; it pertains to social values and standards, which are enforced *via* informal institutions. In the current research, the normative dimension refers to how members of the community view entrepreneurs and how the media plays a role in the startup process. Finally, the cultural-cognitive dimension is defined as a shared understanding of the nature of social reality and the frames in which meaning is created (Scott, 2014). In this work, the cultural-cognitive dimension concerns the ways in which people’s thoughts and beliefs as well as their interactions with other entrepreneurs shape their desire to start a firm (Arenius and Minniti, 2005). Accordingly, we built on institutional theory to investigate the impact of selected institutions on establishing new businesses in Saudi Arabia.

### Regulative Dimension

#### Business Legislations and Policies and Small Business Creation

Laws, regulatory standards and state policies that support new enterprises, minimize risks for those who establish small businesses, and make it easier for business owners to obtain resources are enacted *via* the regulative dimension (Urbano and Alvarez, 2013). The various components of the regulative dimension have been reported in the existing literature to play a critical role in motivating entrepreneurs and stimulating the entrepreneurship process, particularly preparing the market for the entry of promising persons (Stephen et al., 2005; Urbano et al., 2018). For example, the implementation of encouraging and appropriate business rules and policies is meant to enhance entrepreneurial activity (Ram et al., 2017) and thereby lead to the creation of new businesses, which in turn results in new job opportunities and accompanying economic growth and development (Stephen et al., 2005; Urbano et al., 2018; Alshebami and Seraj, 2021a). In contrast, countries that impose high taxes and additional business laws and restrictions, particularly concerning the labor force, discourage people from starting small businesses and have a negative impact on entrepreneurship (Van Stel et al., 2007; Raza et al., 2018). States with effective governmental laws, on the other hand, instill an entrepreneurial mindset that supports new business ventures and enhances the development of existing enterprises while supplying both with the necessary resources (Boudreaux et al., 2019; Camelo-Ordaz et al., 2020). Thus, based on the preceding explanation, we assumed:

H1: *The presence of inadequate governmental regulations and policies contributes negatively to the establishment of small businesses in Saudi Arabia.*

### Normative Dimension

#### Media Attention and Small Businesses Creation

The normative dimension reveals people’s genuine appreciation and respect for entrepreneurs and whether they think starting a business is a desirable career choice. This dimension encompasses many facets of entrepreneurship, including how society views entrepreneurs and the media attention entrepreneurs receive. This article focuses on the effect of media attention on the creation of small businesses. Accordingly, the extant literature emphasizes that the more media attention is given to entrepreneurship, the more entrepreneurship grows (Urbano and Alvarez, 2013; Achtenhagen, 2017; Labaf and Williams, 2018). Media attention also plays an essential role in supporting entrepreneurs, particularly female small business owners (Al-dajani and Marlow, 2010; AbuBakar et al., 2017; Aljarodi, 2020). Media attention is considered significant for business creation because of its influential role in the spread of knowledge, business information, new ideas, success stories, sources of resources and news about relevant institutional policies and opportunities (de Pillis and Reardon, 2007; Urbano and Alvarez, 2013; Ahmed et al., 2019). Furthermore, researchers have examined the significant role of media attention in gender socialization in families, among friends and at school (Eddleston and Powell, 2008). Therefore, we based on the preceding discussion, we formulated the following hypothesis:

H2: *The presence of adequate media attention contributes positively to establishing small businesses in Saudi Arabia.*

#### Entrepreneurs’ High Status and Small Business Creation

According to the definition of high status, successful entrepreneurs in a particular society are accorded high status and respect. This demonstrates the extent to which people in a given society value and appreciate innovative thinking and entrepreneurial endeavors (Busenitz et al., 2000; Urbano and Alvarez, 2013). In the process of launching a business, status, prestige and lifelong learning are all regarded to play a role. Even though status has been established as a crucial notion, conventional economics has been slow to adopt it (Kwon and Milgrom, 2007). It has been observed that there are societies that revere cultural heroes, which includes—and therefore motivates—entrepreneurs (Malach-Pines et al., 2005); other societies, however, do not appreciate entrepreneurs in this way (Dana and Peterborough, 1986). The extant literature on status and its impact, particularly on individual behavior in the job market, is limited (Grund and Sliwka, 2005; Parker and van Praag, 2010). In the context of the current study and according to a 2016 GEM poll, two-thirds of respondents in the Arab world viewed entrepreneurs as high-status individuals (Aminova et al., 2020). Thus, we hypothesize:

H3: *The view of entrepreneurs as having a high status contributes positively to establishing small businesses in Saudi Arabia.*

### Cultural-Cognitive Dimension

#### Fear of Failure and Small Business Creation

The fear of failure in the ambiguous and unpredictable performative area of entrepreneurship is described as an adverse emotional reaction based on cognitive judgments of the risk of failure (Cacciotti et al., 2020). Individuals’ feelings of failure can produce a sense of helplessness when attempting to start a new business (Ajzen, 1991). Those individuals who can control or minimize the fear of failure may therefore be able to lessen the sense of helplessness (Gilmore et al., 2004). Furthermore, subjective thoughts, perceptions and opinions regarding specific events in the entrepreneurial process can impact entrepreneurial activity (Urbano and Alvarez, 2013). For example, individuals who have a favorable view of themselves and are confident in their abilities and knowledge have a greater likelihood of seizing and fully benefiting from business opportunities (Shane, 2000). People, in general, should be able to overcome their fear of failure in order to take full advantage of available opportunities. The capacity to take risks and the capability to deal with uncertainty are commonly recognized as two fundamental skills that distinguish entrepreneurs from non-entrepreneurs (Begley, 1995; Cromie, 2000; Thomas and Muller, 2000). Accordingly, the following hypothesis was developed:

*H4*: *Individuals’ feelings of failure have a negative impact on the establishment of small businesses in Saudi Arabia.*

## Research Methodology

### The Conceptual Model

**FIGURE 1:**
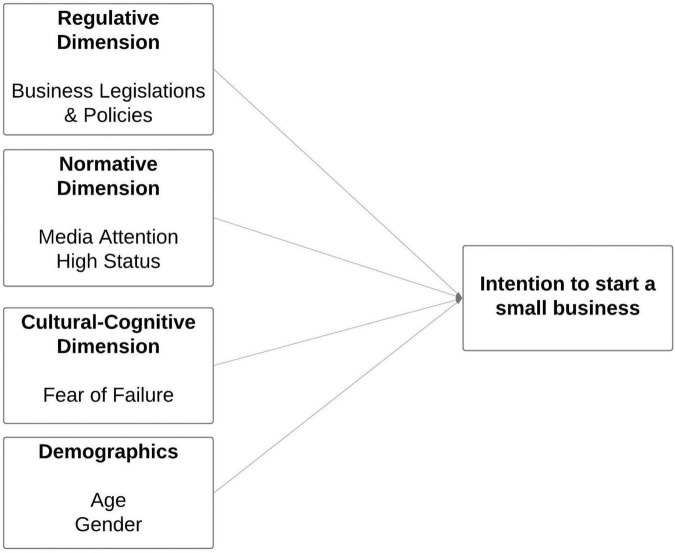
Hypothesized Model. Source: Author’s elaboration.

Figure 1 depicts the conceptual model used to investigate the impact of selected regulative (business legislations and policies), normative (media attention and high status) and cultural-cognitive (fear of failure) factors on the formation of small businesses in Saudi Arabia. To support the relationships in the hypothesized model and limit the likelihood of error, the model included age and gender as control variables.

### The Study’s Measures Respondents

This study was quantitative in nature, relying entirely on secondary data collected by the GEM in 2016 through the General Adult Population Survey (APS), which collected cross-sectional data from 3,376 respondents. The survey included many questions from which the measures for the present research were chosen. For the purposes of this study, the respondents were regarded as nascent entrepreneurs.

### Description of Study Constructs

Table 1 outlines the different constructs assessed in the study: the intention to start a small business (dependent variable) and business legislations and policies, media attention, high status, and fear of failure (independent variables). The study also included some control variables, i.e., age and gender. The aim here was to explore how the selected independent variables influenced the decision of Saudi individuals to start a small business.

**TABLE 1:** Description of constructs.

Dependent variable	Description	
Intention to start a small business	Respondents were asked about their intention to start a business in the next 3 years.	Yes = 1 No = 0
**Independent variables**		
Business legislations and policies	Respondents were asked about the ease with which a small business can be started in Saudi Arabia.	Yes = 1 No = 0
Media attention to entrepreneurs	Respondents were asked whether the media in Saudi Arabia gives more attention to successful entrepreneurs and entrepreneurship.	Yes = 1 No = 0
High status of entrepreneurs	Respondents were asked whether successful entrepreneurs receive respect and are afforded high status in Saudi Arabia.	Yes = 1 No = 0
Fear of failure	Respondents were asked whether fear of failure would prevent them from starting a small business in Saudi Arabia.	Yes = 1 No = 0
**Control variables**		
Gender	Gender	Male = 1 Female = 2
Age	Age of respondents	Birth year

*Source: Author’s elaboration.*

### Descriptive Statistics of the Study Measures

According to the results of Table 2, 76% of the respondents thought that starting a new business in Saudi Arabia was easy, and 79% of the same respondents felt that media exposure helped successful entrepreneurs. Furthermore, 80% of respondents agreed that entrepreneurs have high status and respect in Saudi society, and 41% of the same respondents admitted to experiencing the fear of failure while establishing their firm. Regarding the intention to start a small business, 27% of the respondents claimed that they planned to do so in the next 3 years. Finally, the average age of the respondents was 35.24.

**TABLE 2:** Descriptive statistics of study measures.

Description	*N*	Minimum	Maximum	Mean	Std. Deviation
Respondents’ opinions on how easy it is to start a small business in Saudi Arabia.	3376	0	1	0.76	0.430
Respondents’ views on the role of media attention in promoting successful entrepreneurs.	3376	0	1	0.79	0.407
Respondents’ perceptions of the prevalence of high status and respect for successful entrepreneurs.	3376	0	1	0.80	0.402
Fear of failing and beginning a business as perceived by respondents.	3376	0	1	0.41	0.492
Respondents’ opinions on the feasibility of starting a small business.	3376	0	1	0.27	0.443
Gender	3376	1	2	1.45	0.497
Age	3376	18	64	35.24	10.840
Valid N (list wise)	3376				

*Source: Author’s elaboration.*

## Results

Table 3 reveals a negative association between inadequate business legislation and policies and the intention to start small businesses in Saudi Arabia. Furthermore, it was discovered that sufficient media attention to successful entrepreneurs positively contributes to the formation of small enterprises. Additionally, the conferral of high status and respect to successful entrepreneurs was found to not have a positive relationship with the decision to start a small business. Moreover, no negative correlation was found between the fear of failure and the formation of small businesses. Lastly, age was found to have a positive relationship with the development of small businesses, although no such connection was revealed in terms of gender.

**TABLE 3:** Logistic regression (DV = intention to start a small business).

	B	S.E.	Wald	*df*	Sig.	Exp(B)
Business legislations and policies	−0.288	0.092	9.858	1	0.002	0.750
Media attention to entrepreneurs	0.187	0.103	3.259	1	0.071	1.205
High status of entrepreneurs	0.073	0.102	0.515	1	0.473	1.076
Fear of failure	0.086	0.083	1.082	1	0.298	1.090
Age	0.016	0.004	19.675	1	0.000	1.016
Gender	0.127	0.081	2.467	1	0.116	1.135
Constant	−1.781	0.216	68.105	1	0.000	0.169

*Source: Logistic regression.*

Table 4 outlines the correlations among the study constructs.

**TABLE 4:** Correlation matrix.

	Constant	Fear of failure	High status	Media attention	Business legislations and policies	Age	Gender
Constant	1.000						
Fear of failure	−0.073	1.000					
High status	−0.269	−0.144	1.000				
Media attention	−0.290	−0.008	−0.213	1.000			
Business legislations and policies	−0.228	0.135	−0.023	−0.223	1.000		
Age	−0.621	0.072	0.002	−0.004	0.019	1.000	
Gender	−0.551	−0.213	−0.004	0.113	−0.041	0.017	1.000

*Source: Logistic regression.*

Table 5 provides the omnibus test results, demonstrating the model’s validity; if the omnibus values were less than 0.05, then the model would be accepted with a satisfactory fit. The findings revealed that the values were indeed all less than 0.05, indicating the satisfactory fit of the model.

**TABLE 5:** Omnibus tests of model coefficients.

		Chi-square	*df*	Sig.
Step 1	Step	36.392	6	0.000
	Block	36.392	6	0.000
	Model	36.392	6	0.000

**Model summary**				
**Step 1**	−2 Log likelihood		Cox and Snell R Square	Nagelkerke R Square
	3,888.738^a^		0.011	0.016

*^a^Estimation terminated at iteration 4 because parameter estimates changed by less than 0.001.*

*Source: Analysis.*

The Hosmer and Lemeshow test was used to confirm the model’s strong fit. Table 6 demonstrates that the significance value of the Hosmer and Lemeshow test was more significant than 0.05, indicating the applicability and fit of the model.

**TABLE 6:** Hosmer and lemeshow test.

Chi-square	*df*	Sig.
14.046	8	0.081

*Source: Analysis.*

## Discussion

The importance of institutions, regardless of their nature, in affecting the entrepreneurial behavior of individuals, either negatively or positively, has been noted repeatedly in the existing literature (North, 1990; Scott, 1995). Nevertheless, as most prior research has been undertaken in affluent countries, it was necessary to perform the same investigation in underdeveloped countries (Zeidan and Bahrami, 2011). Thus, the impact of the chosen institutions on the establishment of small businesses in Saudi Arabia was explored in present work, with a variety of outcomes.

The first hypothesis concerned the regulative dimension, i.e., Saudi Arabia’s business laws and policies and their relationship to the establishment of businesses in Saudi Arabia, and revealed a negative correlation (β = −0.288, *P* < 0.05). This is clear since the presence of insufficient business laws and policies discourages people in general, and entrepreneurs in particular, from beginning or expanding their businesses by 0.750 times. This result supports the first hypothesis, consistent with earlier research (Stephen et al., 2005; Van Stel et al., 2007; Urbano and Alvarez, 2013; Ram et al., 2017; Aljarodi, 2020).

In addition, hypotheses relating to the normative dimension, i.e., media attention to successful entrepreneurs and Saudi society’s conferral of high status to entrepreneurs, yielded intriguing results. The presence of adequate media coverage of entrepreneurs was found to have a significant effect (β = 0.187, *P* < 0.10). To put it another way, the more media attention is given to successful businesses, the more entrepreneurs are created, by 1.205 times. These findings are consistent with those of Urbano and Alvarez (2013). In contrast, the connection between high status and entrepreneurs in Saudi society was found to not be significant (β = 0.073, *P* > 0.05), which contradicts the conclusions of previous studies (Aidis et al., 2008; Urbano and Alvarez, 2013; Aljarodi, 2020). This could be due to the prevalence of traditional Saudi culture, values and perceptions, which tend to negatively regard the establishment of small enterprises and the pursuit of government jobs.

Likewise, the cultural and cognitive dimension of the fear of failure was investigated, with the findings revealing no significant negative link to starting a business (β = 0.086, *P* > 0.05). The results for this hypothesis also contradict those of prior investigations (Noguera et al., 2013; Urbano and Alvarez, 2013; AbuBakar et al., 2017). This could be attributable to the fact that Saudi young are willing to take greater risks to gain meaningful employment, especially in light of the government’s new, rigorous laws on employment in the public sector. This might be interpreted as Saudi entrepreneurs being younger and more enthusiastic, especially as 50% of the Saudi population are young, i.e., under the age of 29 (Nieva, 2015). Thus, they are ready to take greater risks. In terms of the control factors, the respondents’ age was shown to have a significant relationship with the decision to start a business (β = 0.016, *P* < 0.05), which is consistent with the findings of Urbano and Alvarez (2013). This could mean that the younger an entrepreneur is, the more interested he or she is in starting a business. Finally, gender had no significant link with the decision to start a small business (β = 0.127, *P* > 0.05), which contradicts previous findings (Le and Minniti, 2006; Urbano and Alvarez, 2013; Aljarodi, 2020).

### Implications

The present study was one of the first to examine the impact of institutions—namely their regulative, formative and cultural-cognitive dimensions (Scott, 1995)—on the establishment of small enterprises in Saudi Arabia. By utilizing GEM data and selecting a sample of relevant institutions for evaluation, this study contributes to the extant empirical literature on institutions and entrepreneurship. More precisely, the present research focused on how these institutions can influence individuals’ behavior, positively or negatively, with respect to establishing a small business (Chew et al., 2021). This is because the impact of these institutions has mostly been overlooked or only minimally touched upon in the existing literature at the national level (AbuBakar et al., 2017). Accordingly, this work considered both males and females at the national level and advanced the application of institutional theory and the entrepreneurship process in Saudi Arabia to assess its national relevance (see Thornton and Ribeiro-Soriano, 2011). The study’s findings in light of Saudi Vision 2030 provide policymakers with guidance for implementing the policies required to foster entrepreneurship in Saudi Arabia. The findings also emphasize the need to devote more media attention to spreading essential information and news about available resources (financial and technical) and successful stories of entrepreneurs to encourage other individuals in the country to launch new businesses (Al-dajani and Marlow, 2010; AbuBakar et al., 2017) and, consequently, reduce the country’s high unemployment rate. Furthermore, the study findings highlight that, despite ongoing efforts by the Saudi government and other official bodies to make the process of starting a business more accessible, challenges remain that prevent some individuals from starting their small businesses, particularly those related to government regulations (Khan and Alsharif, 2019).

Furthermore, the lack of an association between fear of failure and the decision to establish a business among Saudi citizens can be attributed to the willingness of Saudi youth to take greater risks and launch a business in an uncertain economic environment, especially given that nearly 50% of the Saudi population are under 29 years of age (Nieva, 2015). This should prompt the Saudi government to appeal to these individuals’ passions and provide them with the necessary financial and technical assistance, such as financing, incubation services and business consulting. Furthermore, in terms of Saudi society’s perceptions of entrepreneurs and their support, it was shown that there is a need to focus on spreading information and awareness about the importance of entrepreneurship and the country’s SME sector. Likewise, there is also a need to instill in Saudi citizens a work culture that will encourage them to start small businesses rather than relying on family assistance (Alshebami and Seraj, 2021b) or government positions and grants.

## Conclusion

The goal of this study was to examine the impact of selected institutions (in terms of business legislations and policies, media attention, entrepreneurs’ status, and fear of failure) on the decision to start a small business in Saudi Arabia. The study used institutional theory, dividing institutions into three types (regulative, normative and cultural-cognitive), and GEM data from 3,376 male and female respondents. The research adds to existing literature by providing empirical evidence on the influence of the selected institutions on the potential for launching new small businesses in Saudi Arabia. The research revealed intriguing findings that supported the hypothesis that there is a negative relationship between business legislations and policies and the ambition to establish a new small business. Furthermore, the results revealed a positive association between media attention and the establishment of businesses. Surprisingly, expectations concerning the fear of failure and high status among successful entrepreneurs were false. Furthermore, the control variables of age and gender were discovered to have varied effects on the respondents’ entrepreneurial intentions. The study recommends giving more attention to institutions in the country and minimizing their adverse effects by creating an encouraging, supportive and rewarding atmosphere for those in the SME sector to stimulate and reinforce creativity, innovation and performance. Finally, the narrow scope of the research and its focus on only a single country may be considered limitations. As a result, future research should more closely examine a greater diversity of institutions within other Gulf countries and conduct inter-institutional and inter-national comparisons. Such research could also assess the impact of institutions on potential entrepreneurs, such as students or those colloquially considered part of Generation Z.

## Data Availability Statement

The original contributions presented in this study are included in the article/supplementary material, further inquiries can be directed to the corresponding author.

## Author Contributions

Both authors listed have made a substantial, direct, and intellectual contribution to the work, and approved it for publication.

## Conflict of Interest

The authors declare that the research was conducted in the absence of any commercial or financial relationships that could be construed as a potential conflict of interest.

## Publisher’s Note

All claims expressed in this article are solely those of the authors and do not necessarily represent those of their affiliated organizations, or those of the publisher, the editors and the reviewers. Any product that may be evaluated in this article, or claim that may be made by its manufacturer, is not guaranteed or endorsed by the publisher.
